# Seroprevalence of adeno-associated virus types 1, 2, 3, 4, 5, 6, 8, and 9 in a Basque cohort of healthy donors

**DOI:** 10.1038/s41598-024-66546-4

**Published:** 2024-07-10

**Authors:** Miguel Navarro-Oliveros, Ander Vidaurrazaga, Gabriel Soares Guerra, Donatello Castellana, Nieves Embade, Oscar Millet, Urko M. Marigorta, Nicola G. A. Abrescia

**Affiliations:** 1 Structure and Cell Biology of Viruses Lab, Center for Cooperative Research in Biosciences (CIC bioGUNE), Basque Research and Technology Alliance (BRTA), Bizkaia Technology Park, 48160 Derio, Spain; 2 Research and Development, CIC bioGUNE, BRTA, Bizkaia Technology Park, Building 801A, 48160 Derio, Spain; 3 Precision Medicine and Metabolism Laboratory, CIC bioGUNE, BRTA, Bizkaia Technology Park, 48160 Derio, Spain; 4 Integrative Genomics Lab, CIC bioGUNE, BRTA, Bizkaia Technology Park, Derio, Basque Country Spain; 5 Basque Foundation for Science, IKERBASQUE, 48009 Bilbao, Spain; 6 Centro de Investigación Biomédica en Red de Enfermedades Hepáticas y Digestivas (CIBERehd), Instituto de Salud Carlos III, Madrid, Spain; 7 Present Address: Central European Institute of Technology, Masaryk University, Brno, Czech Republic

**Keywords:** Adeno-associated virus, Seroprevalence, Gene therapy, Basque population, Epidemiology, Gene therapy, Genetic vectors

## Abstract

Adeno-associated viruses (AAVs) are promising gene therapy vectors, but challenges arise when treating patients with preexisting neutralizing antibodies. Worldwide seroprevalence studies provide snapshots of existing immunity in diverse populations. Owing to the uniqueness of the Basque socio-geographical landscape, we investigated the seroprevalence of eight AAV serotypes in residents of the Basque Country. We found the highest seroprevalence of AAV3, and the lowest seroprevalence of AAV9. Additionally, less than 50% of the Basque population has neutralizing antibodies against AAV4, AAV6, and AAV9. Our findings provide insight into AAV infections in the Basque region, public health, and the development of AAV-based therapeutics.

## Introduction

Adeno-associated virus (AAV) is a nonenveloped, single-stranded DNA virus belonging to the *Parvoviridae* family (genus *Dependoparvovirus*). Structurally, virions assemble into an icosahedral particle with a diameter of approximately 250 Å. A fully permissive infection requires the presence of an adenovirus or herpes simplex virus^1^. Since AAV is non-pathogenic to humans, AAV vectors are typically used in gene therapies that are directly administered to patients by infusion or local administration (in vivo). As of April 2024, the U.S. Food and Drug Administration (FDA) has approved six gene therapy medicines based on AAV vectors, e.g., BEQVEZ®, ELEVIDYS®, HEMGENIX®, LUXTURNA®, ROCTAVIAN® and ZOLGENSMA®. Limitations of AAV vector treatment include overcoming the immune system and unloading the transgene cargo. Preexisting immunity poses a significant challenge for gene therapies that use viral vectors, especially for AAV; since they are harmless, AAVs are commonly present in the population^2–4^. Various strategies are being developed to make the AAV carrying the repairing gene immune system-invisible, employing techniques such as haploid capsid engineering, chemical conjugation, or suppressing the recipient's immune response with immunosuppressor drugs^5–8^. Thirteen human/nonhuman primate AAV serotypes have been identified (and more than 100 variants), and not only do each of them display a distinct cell tropism but they also show a different seroprevalence and geographic distribution in the population^9,10^. Thus, fulfilling the concept of personalized medicine, potential patients are screened for the presence of preexisting antibodies against the different serotype viral capsids so as to increase patient care and the effectiveness of treatment within an optimal age window^11^. Population assessment of anti-AAV immunity is therefore necessary for obtaining clinical data and for developing new viral capsids. Several seroprevalence studies worldwide, including in the U.S., Europe, Africa, Asia, and Australia, have been carried out to determine the proportion of individuals with neutralizing antibodies (NAbs) against AAVs^10,12–15^. The gathered insights provided relevant information on which serotypes are more commonly circulating across different locations worldwide.

The Basque Country is a region that spans parts of northern Spain and southwestern France. In recent years, autochthonous Basque inhabitants have garnered significant research attention due to their distinctive cultural, biological and genetic background from other European populations^16,17^. However, according to the Basque Institute of Statistics (EUSTAT), as of January 2022, nearly 30% of the current residents of the Basque Country were born in the remaining Spanish provinces and to foreigners (https://en.eustat.eus/indice.html).

We sought to target a healthy cohort of Basque country residents for the presence of NAbs against AAVs to assess their frequency and cross-reactivity. We showed that neutralizing antibodies against AAV3 were the most prevalent antibodies in our cohort. In addition, using a 95% confidence interval, we extrapolated that less than 50% of Basque residents possess NAbs against the AAV4, AAV6 and AAV9 serotypes. Finally, it is possible to statistically detect differences in the NAb seroprevalence for the AAV5, AAV8 and AAV9 serotypes in the European/Mediterranean landscape, even though the prevalence of these serotypes was opposite to that in countries geographically close such as France or Italy.

## Results

### Seroprevalence of anti-AAV NAbs in the Basque cohort

**Figure 1:**
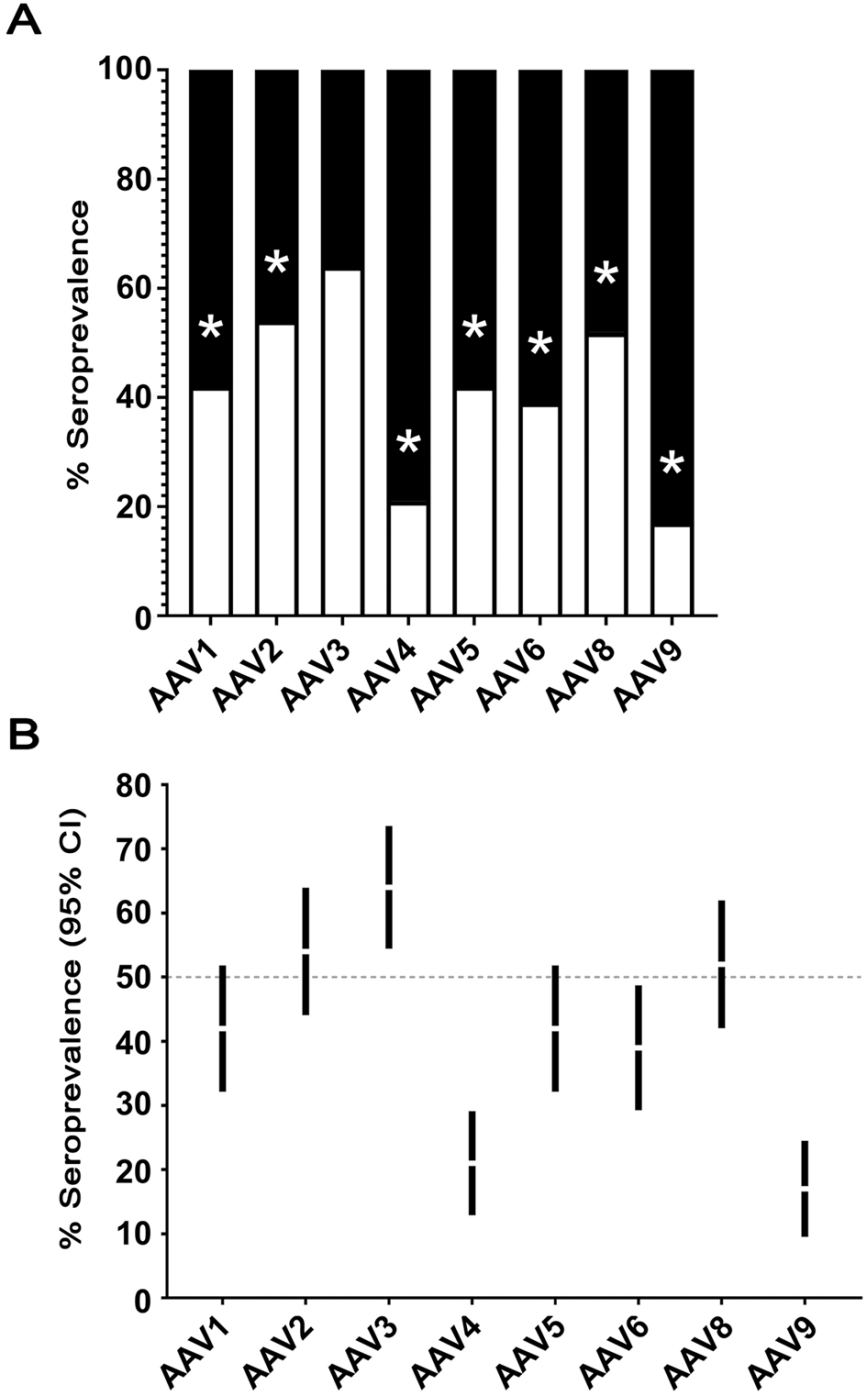
**A** Seroprevalence of neutralizing antibodies (NAbs) against different adeno-associated virus (AAV) serotypes in 100 healthy human serum samples from residents of the Basque Country. Samples were considered positive if the 1:20 serum dilution reduced the vector transduction ≥ 50%. The asterisk indicates statistical significance (*p* value ≤ 0.05). **B** 95% confidence intervals (CIs) for neutralizing antibodies against AAVs within the Basque population.

Serum samples from 100 healthy residents of the Basque Country were analysed for the presence of antibodies against AAV serotypes 1, 2, 3, 4, 5, 6, 8, and 9. NAbs were considered positive if a serum dilution of 1:20 was able to inhibit vector transduction by ≥ 50%. In our cohort, the seroprevalence was highest for AAV3 (64%), followed by AAV2 (54%), AAV8 (52%), AAV1 and AAV5 (42%), AAV6 (39%), AAV4 (21%) and finally AAV9 (17%). Compared to the AAV3 percentage, a significant reduction in seroprevalence was reported for all serotypes (Fig. 1A).

Considering the average and 95% confidence intervals, we concluded that less than 50% of people had NAb to AAV4, AAV6 or AAV9 (Fig. 1B). Furthermore, we did not observe any gender dependency in the seroprevalence (*p* values: 0.05 for all tests).

### Co-seroprevalence of neutralizing antibodies

Seroreactivity between AAV serotypes provides valuable information for inferring which serotype would be more advantageous for development or administration than other serotypes. All sera positive for NAbs against AAV1 showed correlation with almost all other serotypes. All samples that were NAbs-positive against AAV1 were also NAbs-positive against AAV2. Furthermore, samples NAbs-positive against AAV1 had a 97.6% chance of having NAbs against AAV3, 83.3% against AAV6, 76.2% against AAV5 and 71.4% against AAV8. A correlation of 31% in NAbs presence was found between AAV1 and AAV9.

Sera NAb positive against AAV2 were also positive for AAV3 (88.9%), AAV1 (77.8%), AAV8 (68.5%) and AAV6 (66.7%); in contrast, only sixteen and fourteen serum samples cross-neutralized the AAV4 and AAV9 serotypes (29.6% and 25.9%). Forty-eight (75%) and forty-two (65.6%) sera NAb positive against AAV3 were also positive against AAV2 and AAV8, respectively. Only fourteen sera (21.9%) positive for AAV3 were found to be positive against AAV4 and AAV9. Of note is AAV4, the serotype with the lowest correlation, with only sixteen sera (76.2%) that also containing NAbs against AAV2. AAV5 co-seroprevalence was detected in forty-two samples. Of these, thirty-six sera (85.7%) were positive for NAbs against AAV3, thirty-four (81%) against AAV2, thirty-two (76.2%) against AAV1 and thirty (71.4%) against AAV6. Lower co-seroprevalences of 31% and 26.2% were found with AAV4 and AAV9, respectively. Remarkably, we found that all sera with AAV6 seroprevalence cross-neutralized the AAV3 serotype. Furthermore, co-seroreactivity of AAV6 NAbs-positive sera was observed with AAV2 (92.3%), AAV1 (89.7%), AAV8 (79.5%), and AAV5 (76.9%), with lower percentages noted for AAV4 (30.8%) and AAV9 (28.2%). NAbs-positive sera against AAV8 showed co-seroreactivity against AAV3 and AAV2 in the 80.8% and 71.2% of cases, respectively, while only 17.3% showed reactivity against AAV4 and AAV9 serotypes.

**Figure 2:**
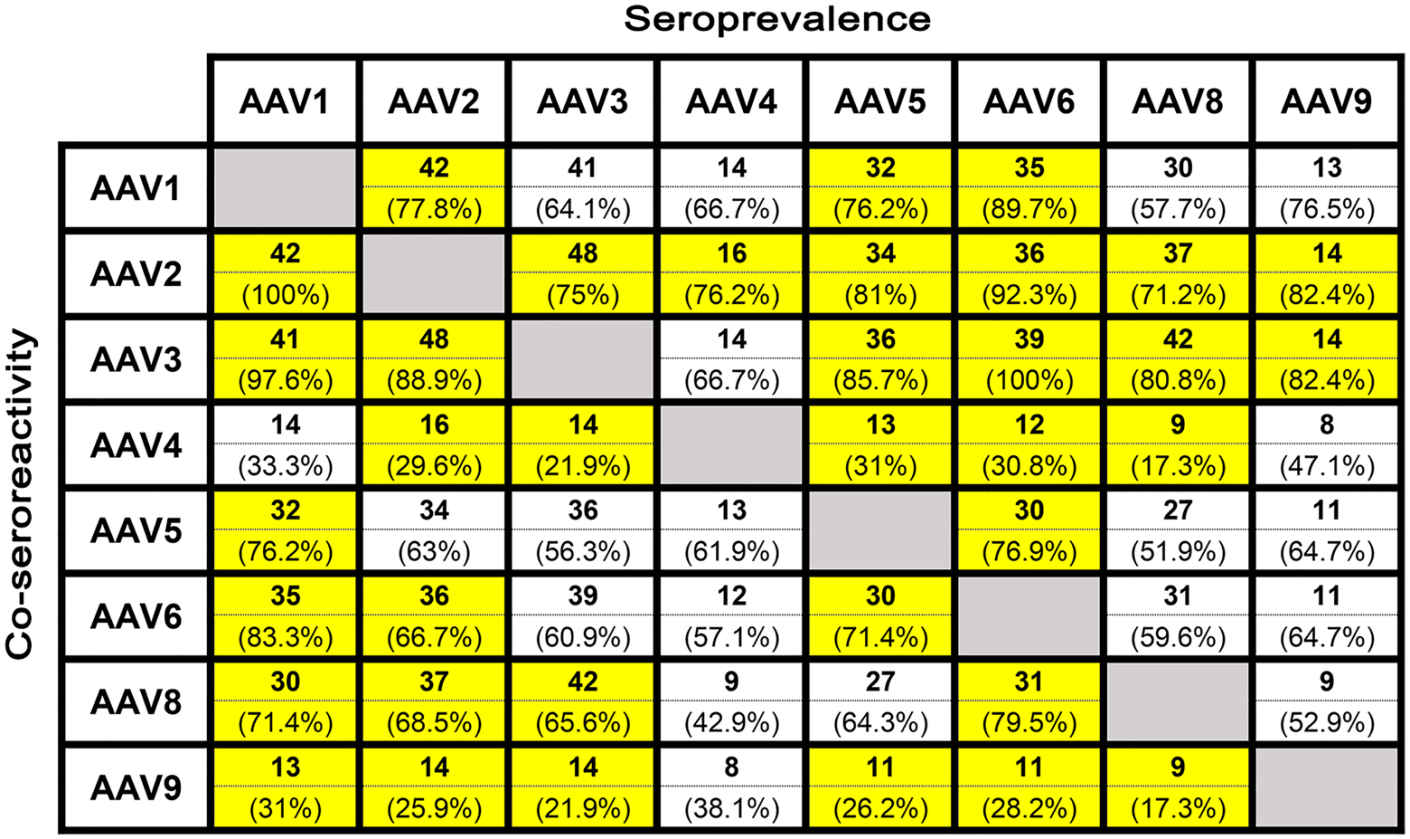
Co-seroprevalence distribution of NAbs against AAV serotypes. The number in bold at the top of each cell indicates the number of sera positive for the serotype in the row among the resulting NAb-positive samples against the serotype in the column. The number in parenthesis at the bottom of each cell indicates the percentage of sera positive against the serotype in the row relative to the total number of NAb-positive sera against the serotype in the column. False discovery rate (FDR) corrected by the Benjamini–Hochberg method (cells in yellow: *p* value ≤ 0.05).

Finally, only two serotypes, AAV2 and AAV3, were neutralised with fourteen serum samples (82.4%) that were NAb-positive against AAV9. The above results are summarised in Fig. 2.

### Comparison of seroprevalence between the Basque cohort and cohorts from European countries

**Figure 3:**
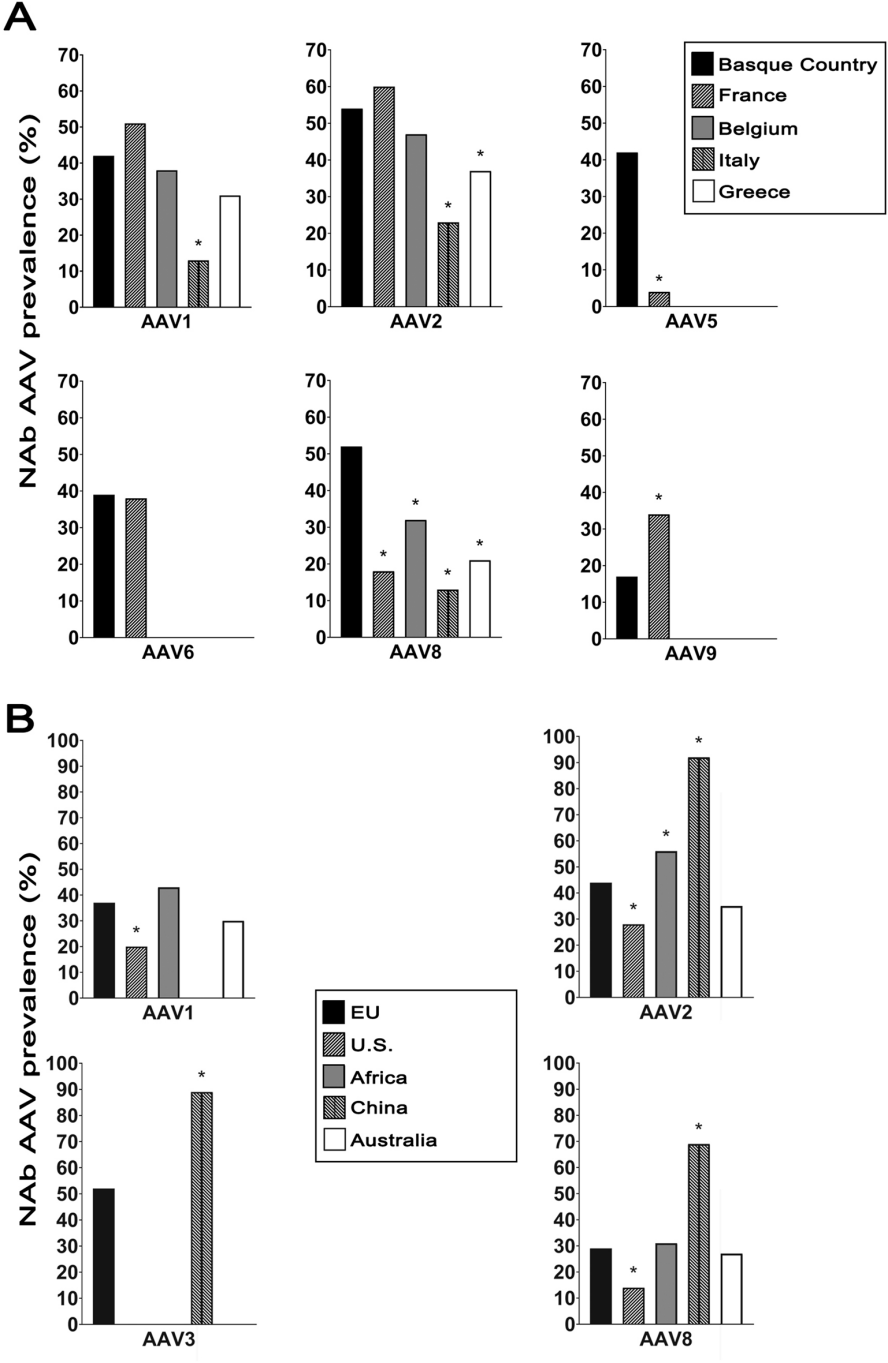
**A** Comparative seroprevalence of NAbs against AAV serotypes across European countries (up to 152 serum samples from France, 100 serum samples from Belgium, 100 serum samples from Italy and 81 serum samples from Greece; data extracted from Refs.^10,13^). The absence of panels for serotypes AAV3 and AAV4 is due to the unavailability of seroprevalence data. The asterisk marks the statistical significance when compared with the same NAb prevalence serotype from the Basque cohort (*p* value ≤ 0.05). **B** Seroprevalence profile of NAbs against different adeno-associated virus (AAV) serotypes in a worldwide comparison to date. The European cohort included 533 serum samples, the African cohort 407, and the U.S., Chinese and Australian cohorts each included 100 serum samples (data extracted from Refs.^10,13,14^). The absence of panels for serotypes AAV4, AAV5, AAV6 and AAV9 is due to the unavailability of seroprevalence data. The asterisk indicates statistical significance compared to the corresponding NAb incidence serotype from the European cohort (*p* value ≤ 0.05).

Previous studies have provided an epidemiological map of NAbs against AAVs in ten different countries across four continents, although not all using the eight serotypes as in our study^10,13^. We compared the seroprevalence observed in the Basque cohort (*n* = 100) with that reported in France (*n* = 152), Belgium (*n* = 100), Italy (*n* = 100) and Greece (*n* = 81). These studies, as well as those worldwide were performed with equivalent serum dilution (1:20)^10,13^. To the best of our knowledge, no seroprevalence study of AAV3 under similar methodological conditions is available for any of the other European cohorts. AAV8 was the only serotype showing a significant difference in seroprevalence when compared to all European cohorts; the Basque cohort showed a 52% NAb seroprevalence, whereas Belgium had 32%, Greece 21%, France 18% and Italy 13%. As previously shown, AAV2 is the serotype that has the highest seroprevalence in the EU and worldwide, whereas in our cohort of Basque residents, AAV3 exhibits the highest seroprevalence. Additionally, our data underline a significant discrepancy in seroprevalence *versus* that found in Greece (37%) and in Italy (23%). Finally, AAV1, AAV5 and AAV9 showed only one country that differed significantly: Italy for AAV1 (13%) and France for AAV5 and AAV9 (4% and 34%, respectively) (Fig. 3A).

### Basque cohort seroprevalence in the context of the worldwide epidemiology of NAbs to AAVs

We updated the European frequencies of AAV seroprevalence with our hundred samples from the Basque cohort and compared them with the available data across countries and continents (*n* = 100 samples for the U.S., China and Australia cohorts; *n* = 407 for the African cohort)^10,14^. The European seroprevalence of anti-AAV1 NAbs (37%) significantly differed from that in the U.S. (20%). Except for Australia, the seroprevalence of anti-AAV2 NAbs (44%) exhibited significant differences from the seroprevalence found in China (92%), Africa (56%) and the U.S. (28%). Moreover, the Chinese cohort showed a large increase in the seropositive profile against AAV3 (52% vs. 89%). Finally, when comparing the AAV8 seroprevalence in the EU (29%), statistically significant differences were found with those in China (69%) and the U.S. (14%) (Fig. 3B).

## Discussion

Adeno-associated viruses have emerged as one of the most promising viral vectors for gene therapy. In the last six years, the U.S. FDA has approved drugs using different serotypes of recombinant AAV vectors, including AAV2, AAV5 and AAV9 (FDA submission tracking numbers 125610, 125694, 125772, 125720, 125781 and 125786, respectively, in chronological order). However, as preexisting NAbs jeopardize effective treatment^18,19^, knowing the population seroprevalence profile has practical value in a clinical setting and for AAV drug development. This study was designed to stratify the seroprevalence of the Basque population. To the best of our knowledge, no prior study of this nature has been conducted in the Basque region. We showed that the population of the Basque Autonomous Region possesses the highest seroprevalence of neutralizing antibodies against AAV3, compared to the AAV2 seroprevalence found in previous reports for cohorts in the U.S., Belgium, Greece, Italy, Kenya, Rwanda, South Africa, Uganda, Zambia, Australia, France, China and Japan^10,12–14,20–22^. In addition, our study revealed that, in the Basque cohort, there was a statistically significant decrease in the AAV1, AAV2, AAV4, AAV5, AAV6, AAV8, and AAV9 seroprevalence *versus* the AAV3 seroprevalence. Moreover, less than 50% of the residents in the Basque Country possess neutralizing antibodies against AAV4, AAV6 and AAV9. In this context, these serotypes might be more suitable targets for gene therapy development.

We also observed co-seroreactivity across different AAV serotypes within the Basque cohort. Co-seroreactivity occurs when (i) an individual has been infected with different AAV serotypes throughout her/his life, thus possessing both specific NAbs, and/or (ii) a NAb raised against a specific serotype recognizes multiple serotypes. The latter difference may be explained by the structural and/or primary sequence homology of the VP1-VP2-VP3 capsid proteins across the different serotypes. An example of this could be those sera positive for neutralizing anti-AAV6 antibodies that are also positive for neutralizing antibodies against AAV1, AAV2 and AAV3 [largest structural pairwise root-mean-square-deviation (rmsd) = 0.8 Å between 518 equivalent Cα in AAV6 versus AAV3 and a minimal sequence identity > 83% between AAV6 versus AAV2; PDB ids for AAV6, AAV1, AAV2 and AAV3: 3OAH, 6JCR, 6IH9 and 3KIE, respectively].

However, this rationale has its limitations as it does not explain the significant differences, such as the low prevalence percentage observed for AAV9 among the AAV8-positive serum samples, despite displaying a rmsd of 1.1 Å between 518 equivalent Cα and a sequence identity > 85% (PDB ids for AAV9 and AAV8: 7MT0 and 6V12, respectively)^23^. It is important to consider that most NAbs interact with the receptor binding domains located at the threefold axis of symmetry on the capsid, and these interactions typically involve a few key residues conformationally competent.

Posttranslational modifications, such as glycosylation, though less common in nonenveloped viruses, remain largely unexplored in AAV research and might modulate NAb recognition^24^.

When analysing our results derived from the Basque cohort against those available from other EU cohorts (France, Belgium, Italy and Greece), we noticed that only seroprevalence data for AAV1, AAV2 and AAV8 were common. A comparison of the percentages of seropositivity against AAV1 showed that in the Basque cohort, there was a statistically significant increase (42%) relative to that in the Italian cohort (13%), which was more in line with the seropositivity values of other EU countries.

In the case of NAbs against AAV2, the Basque cohort (54%) had seroprevalences similar to those of the French and Belgian cohorts but significantly different from the lower percentages of the Italian (23%) and Greek (37%) cohorts. Notably, for the remaining serotypes, when we compared the Basque cohort against the French cohort (which is the closest country geographically), there were large statistically significant differences in AAV5 (42% vs. 4%) and AAV9 (17% vs. 34%)^10,13^.

When all European data were combined, AAV2 (44%) and AAV8 (29%) were the serotypes that exhibited the most significant differences, with a decrease in prevalence for both serotypes in the U.S. (28% and 14%), an increase in both serotypes in China (92% and 69%, respectively) and an increase in AAV2 in Africa (56%). Regarding the prevalence of AAV1, only the U.S. (20%) showed a decrease when compared to Europe (37%). Finally, only one previous study analysed NAbs against the AAV3 serotype, revealing that there is a large statistical increase in NAbs in China (89%) compared to Europe (52%). Intriguingly, Australia, the furthest location from Europe, showed no significant differences from the EU cohort in any of the serotypes analysed.

In conclusion, our AAV seroprevalence study in a cohort of residents of the Basque Country provides an epidemiological picture that supports a seroprevalence profile closer to that of countries geographically located in Western Europe rather than in Southern Europe. Nevertheless, as in many countries worldwide, the Basque region is experiencing transformation due to widespread migration movements. Consequently, these studies require constant updates to determine the evolving seroprevalence profile of NAbs against AAVs worldwide, which is crucial for developing AAV-based gene therapies.

## Methods

### Adeno-associated virus serotypes

AAV-CMV vectors (serotypes 1–6, 8, and 9) harboring a luciferase reporter gene were purchased from the UNC Vector Core (https://www.med.unc.edu/genetherapy/vectorcore/in-stock-aav-vectors/).

### Source of serum sample

Hundred serum samples from the general working population in the Basque Country (50 females, 50 males, aged 33–60) were obtained from the Basque Biobank (www.biobancovasco.org). According to the Declaration of Helsinki principles, all study participants provided written informed consent for research using an approved consent form, and the study received evaluation and approval from the corresponding Ethic Committee OSARTEN (CEIC-E 16-114). Anonymized samples were collected during the 2018–2019 annual medical check-ups in collaboration with Osarten Kooperatiba Elkartea from Mondragon Corporation.

### Cell culture and neutralization antibody assay

The following cell types were used for the experiments depending on the serotype of rAAV used: HeLa (rAAV1, rAAV2, rAAV5, rAAV6 and rAAV9), HEK293T (rAAV3), COS-7 (rAAV4) or Huh7 (rAAV8)^13,20,25^. The cells were maintained in Dulbecco’s modified Eagle’s medium (DMEM; Lonza, 12-604F) supplemented with 10% fetal bovine serum (FBS; Corning, 35-079-CV) and 100× nonessential amino acid solution (NEA; Lonza, 13-114E) at 37 °C in a 5% CO_2_ atmosphere.

On the initial day, 5 × 10^4^ cells were seeded in each well of a 48-well plate (Nunclon^®^ Delta Surface; Thermo Fisher, 150687) in 200 µL of cell culture media. On day 2, rAAV was diluted in Dulbecco's phosphate-buffered saline (DPBS; Gibco, 14040-091) supplemented with DMEM to achieve a previously determined multiplicity of infection (MOI) for optimal reporter gene transduction^13,20,25^. The following MOIs, expressed as the number of virus genomes (VG) needed to infect individual cells, were used: 3 × 10^3^ VG/cell for rAAV4; 5 × 10^3^ VG/cell for rAAV2, rAAV5 and rAAV6; 10^4^ VG/cell for rAAV1, rAAV8 and rAAV3; and 10^5^ VG/cell for rAAV9. Different MOIs were used based on the previously described virus entry capabilities of each cell type^13,20,25^.

Serum samples were inactivated by a 56 °C heat shock for 30 min followed by dilution in DPBS (dilution 1:20) as previously described^12^. Diluted serum samples and vectors were incubated in a total volume of 33 µL/well (1:1 ratio) for 1 h at 37 °C. Pooled serum from wild-type C57BL/6 mice was mixed with each rAAV serotype to obtain the above MOIs, which were used as a positive control; DPBS-DMEM was used as a negative control instead of rAAV.

Serum-vector mixture was added to each well, followed by 48 h of incubation at 37 °C and 5% CO_2_. Luciferase reporter protein was quantified using a Bright-Glo™ assay (Promega, E2610) in an Optiplate™-96 F (PerkinElmer Life Science, 6005270) with a VICTOR2 microplate luminometer (PerkinElmer Life Science).

The detection of neutralizing IgGs against individual AAV serotypes was considered positive if serum dilutions of 1:20 were able to inhibit vector transduction by ≥ 50% when compared to positive control.

### Statistical analyses

The prevalence of NAbs for each AAV was calculated as the percentage of serum samples positive for IgGs among the total number of available samples (*n* = 100).

To assess the significance of the differences in seroprevalence across serotypes, we employed a chi-square test with AAV3 prevalence as the reference (*p* value ≤ 0.05).

To infer the NAb prevalence within the Basque population, we used the normal approximation to obtain the 95% binomial confidence interval (CI) for each serotype, assigning 1 to the sera when positive for NAbs and 0 to the sera when negative for NAbs.

The significance of the co-seroprevalence analysis was corrected by the Benjamini–Hochberg method to minimize the false discovery rate (FDR). To compare seroprevalences across countries, the same chi-square statistical framework was adopted. We plotted the prevalence values for each serotype of the Basque cohort against the equivalent serotype (when available) in each of the European countries using a chi-squared distribution. The same methodology was followed for the European cohort against other worldwide cohorts. We used a 5% alpha threshold to declare significance. To this end, GraphPad Prism software, version 10.0.2, was used.

## Data Availability

The datasets used and/or analysed during the current study are available from the corresponding author on reasonable request.
